# Less Users More Confidence: How AOIs Don't Affect Scanpath Trend Analysis

**DOI:** 10.16910/jemr.10.4.6

**Published:** 2017-11-22

**Authors:** Sukru Eraslan, Yeliz Yesilada, Simon Harper

**Affiliations:** Middle East Technical University, Northern Cyprus Campus, 99738 Kalkanlı, Güzelyurt, Mersin 10, Turkey; School of Computer Science, University of Manchester, Manchester, M13 9 PL, United Kingdom

**Keywords:** eye tracking, scanpath, usability, sample size, region of interest, areas of interest, visual elements, web pages, segmentation, trending path, STA

## Abstract

User studies are typically difficult, recruiting enough users is often problematic and each experiment takes a considerable amount of time to be completed. In these studies, eye tracking is increasingly used which often increases time, therefore, the lower the number of users required for these studies the better for making these kinds of studies more practical in terms of economics and time expended. The possibility of achieving almost the same results with fewer users has already been raised. Specifically, the possibility of achieving 75% similarity to the results of 65 users with 27 users for searching tasks and 34 users for browsing tasks has been observed in scanpath trend analysis which discovers the most commonly followed path on a particular web page in terms of its visual elements or areas of interest (AOIs). Different approaches are available to segment or divide web pages into their visual elements or AOIs. In this paper, we investigate whether the possibility raised by the previous work is restricted to a particular page segmentation approach by replicating the experiments with two other segmentation approaches. The results are consistent with ~5% difference for the searching tasks and ~10% difference for the browsing tasks.

## Open Data

The dataset used for the experiments is provided in external online repository at http://iamdata.cs.manchester.ac.uk/data_files/33. It consists of the saved versions of the web pages used, information sheet, consent form, questionnaire and individual scanpaths in terms of the AOIs of the web pages. The repository also includes all the raw data from the experiments. Besides, the Python implementation of the Scanpath Trend Analysis (STA) algorithm can be accessed from https://github.com/SukruEraslan/sta.

## Introduction

User studies play an important role in improving user experience on the web. In these studies, eye tracking has been widely used for assessing the quality of user experience on web pages ( 1 ). It has also been used for investigating user interactions with web pages to provide some directions to improve user experience ( 2-4 ). However, as eye tracking researchers, we are commonly having difficulties finding users for our studies ( 5 ). In an eye tracking study, a researcher is required to allocate a separate session for each user, and therefore the study cannot be carried out in parallel with only one eye tracker and/or one researcher. Hence, these studies can take a significant amount of time to be completed. To deal with this issue, it is important to estimate the ideal number of users to understand and model user behaviours on the web. Even though eye tracking is increasingly used in usability studies to evaluate and improve usability of web pages ( 1 ), existing research does not focus on the effects of the number of users on data analysis in this field. Specifically, the number of users required for analysing eye movement sequences (i.e., scanpaths), which is typically conducted based on visual elements or areas of interest (AOIs) of web pages, has not been studied in depth in the literature ( 6 ). Different approaches can be used to divide or segment web pages into their visual elements or AOIs manually or automatically ( 7 ). Researchers can apply an automated approach to discover AOIs of web pages which typically uses the source code with some heuristics. Researchers can also manually define their AOIs based on their goals. Specifically, if they are interested in certain elements on web pages (such as, advertisements), they can directly define them as their AOIs. However, there is no investigation of possible effects of AOIs on the required number of users for scanpath analysis. In the rest of the paper, we will refer to the process of identifying AOIs or visual elements on web pages as segmentation, thus the AOIs or elements will be referred to as segments.

It has been shown that there is a possibility of achieving almost the same results with fewer users, especially achieving 75% similarity to the results of 65 users with 27 users for searching tasks and 34 users for browsing tasks in scanpath trend analysis (STA) ( 6 ). The STA algorithm is designed to identify the most commonly followed path as trending path on a particular web page in terms of the AOIs of the page ( 8 ). The trending path provided by the STA algorithm can be used to improve the web experience of users in constrained environments, especially visually disabled users ( 8 ). In particular, web pages can be adapted for visually disabled users by making commonly used areas more accessible in the desired order such that the users can directly access these areas with their screen readers without spending unnecessary time on clutter ( 4 ).

To investigate the possibility of achieving almost the same results with fewer users in scanpath trend analysis, only the extended and improved version of the Visionbased Page Segmentation (VIPS) approach was used by Eraslan, Yesilada ( 6 ) to automatically segment the web pages into their AOIs ( 9 ). However, the selection of a page segmentation approach may affect the possibility of achieving the same results with fewer users in scanpath analysis. Therefore, in this paper, we replicate the experiments from Eraslan, Yesilada ( 6 ) with the same eye tracking dataset by using different segmentation approaches to investigate whether this possibility is restricted to a particular segmentation approach.

**Figure 1.**
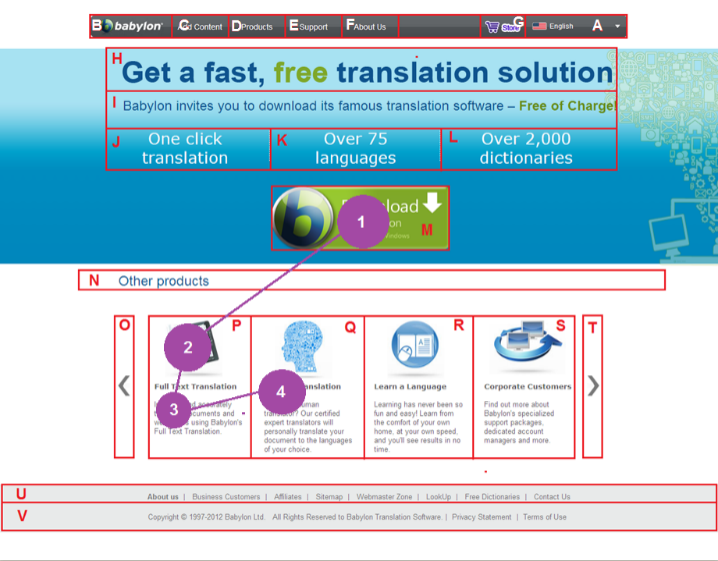
A user scanpath on the Babylon web page that is segmented into its AOIs by the VIPS approach

As an example, Figure 1 shows the Babylon page with its AOIs automatically discovered by the extended and improved version of the VIPS approach ( 9 ). The VIPS approach segments web pages based on both their source code and visual representation, and relates their elements to the underlying source code ( 9 ). Figure 1 also illustrates a scanpath of a particular user on the Babylon page where the circles represent the points fixated by the user and the largest circle shows the longest fixation ( 10 ). As illustrated in the figure, scanpath analysis allows the investigation of which elements catch attention and which paths are followed in terms of the elements ( 8 ).

To investigate the validity of the possibility of achieving almost the same results with fewer users with other page segmentation approaches, we firstly reviewed the literature to find appropriate segmentation approaches for our study. However, we could find only a few public segmentation approaches. This is a common problem in computer science as highlighted by Collberg and Proebsting ( 11 ). Thus, we could replicate the experiments with two other page segmentation approaches. As an alternative to the VIPS approach, we used the Block-oMatic (BOM) approach for the automatic segmentation ( 7, 12 ). The BOM approach also segments web pages based on both their source code and visual representation, but uses an alternative hybrid algorithm to do that. As researchers can also prefer to segment web pages manually based on their goals ( 13 ), in addition to the BOM automatic segmentation approach ( 12 ), we decided to apply a user driven approach to manually segment the web pages into their AOIs. For the manual segmentation, we again used the same dataset ( 6 ) because the participants were asked to draw what they remember about the layout of the web pages on a piece of paper. This method is called recall. As it is explained by Johnson ( 14 ), visual cues are very important to support users to recognise where they are. This recall approach helps us to understand which visual elements/AOIs of the pages really supported that. Those recalled by the participants were more likely to support their tasks. After the participants were asked to draw what they recall, we combined their drawings to manually segment the pages into their elements. We could also segment the web pages manually by ourselves but we used the recall approach to be more objective. If we drew the segments by ourselves, this would reflect our particular view of the segments on the pages which would not be objective. By using the recall method, we discovered how the participants generally segmented the pages in their minds and how the design of the pages supported that. Since they drew the segments based on what they remember about the pages, the drawn segments would have taken their attention, thus could be valuable in scanpath trend analysis.

The rest of this paper firstly discusses the related work, secondly explains our methodology including the STA algorithm, the eye tracking study, the segmentation approaches used and our analysis procedure, then presents the results along with their discussion and finally provides the conclusions.

## Related Work

Usability studies are conducted to evaluate a particular product, such as a web page. Usability experts can observe how users interact with a particular product to complete certain tasks and/or they can examine the product to investigate possible problems that can be experienced by users ( 15 ). The number of users required for usability studies has been a debatable issue for more than 30 years ( 15, 16 ). Specific numbers have already been suggested but these numbers are typically related to the studies which attempt to identify usability problems of a particular product ( 6 ). The most popular example is from Nielsen and Landauer ( 17 ) who suggest that it is possible to discover 85% of usability problems of a particular product with only five users. In contrast, Faulkner ( 18 ) suggests that this is not valid for all five users. Based on her study, 15 users are required instead of five users. Another example is from Hwang and Salvendy ( 19 ) who analysed many published research papers since 1990 and suggest that at least 10±2 users are needed to discover 80% of usability problems. However, there is also a considerable number of researchers who argue that each study has its own features, thus the number of users cannot be unique for all studies ( 15, 20-22 ). For instance, the complexity of products and/or the characteristics of users can cause differences in the required number of users ( 23, 24 ).

**Figure 2.**
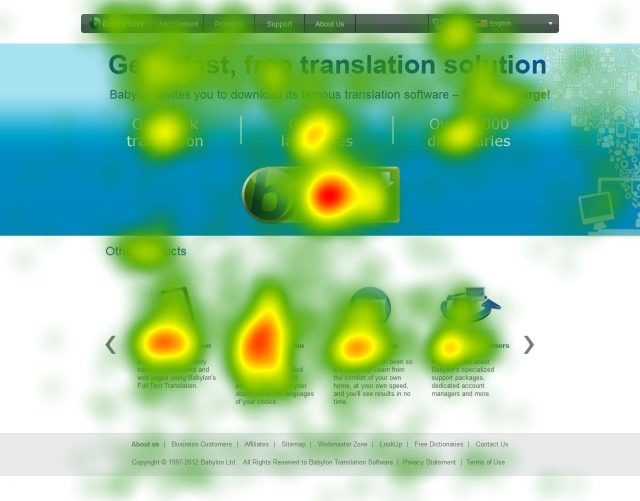
A heat map on the Babylon web page

Existing research in this field does not focus on eye tracking studies which are commonly used to evaluate and improve usability of web pages ( 1 ). Since eye tracking studies are usually time-consuming, it is crucial for researchers to estimate the ideal sample size for their studies. Pernice and Nielsen ( 25 ) suggest to have 39 users for a stable heat map to highlight which parts of web pages get more attention. However, as shown in Figure 2, these maps do not show eye movement sequences and they are much easier to analyse in comparison with scanpaths. Therefore, we should not assume that this number is also appropriate for scanpath analysis. Moreover, Pernice and Nielsen ( 25 ) suggest that a qualitative study can be conducted with six users and then their eye movements can be watched. Although researchers may gain valuable insights for determining which fixation features (such as, fixation duration, fixation count, time to first fixation, etc.) would be relevant for their further analyses by watching eye movements of users, the details are likely to be lost as an eye tracking dataset typically consists of many fixations. Therefore, detailed data analysis is crucial and becomes critical compared to qualitative analysis. Furthermore, eye tracking data analysis also needs to be related to certain behavioural tasks. Otherwise, the analysis cannot be completed to understand the user behaviour.

The effects of the number of users on scanpath analysis were investigated by using a scanpath analysis algorithm called Scanpath Trend Analysis (STA) on six web pages segmented with the VIPS approach ( 6 ). This algorithm discovers the trending scanpath on a particular page in terms of its AOIs ( 8 ). This path is different from an absolute path that is shared by all users. Other existing approaches typically try to discover an absolute path (26). However, their resulting paths tend to be very short because of the variations caused by individual differences, and therefore they typically have low similarities to the user scanpaths ( 8, 26 ). In comparison with other approaches, the STA algorithm provides the path with the highest similarities to the user scanpaths, thus it would be more helpful for behaviour analysis ( 8 ). The details of the STA algorithm is provided in the following section.

The study with the STA algorithm showed that it is possible to achieve almost the same results with a less number of users ( 6 ). Specifically, there is a possibility of achieving 75% similarity to the results of 65 users with 27 users for searching tasks and 34 users for browsing tasks. However, the study did not show whether this possibility is valid with other page segmentation approaches. Thus, we ask whether it matters how we segment a web page.

## Methodology

This section firstly gives a brief information about the STA algorithm, secondly explains our eye tracking study, and then describes the segmentation approaches used and our analysis procedure.

### STA: Scanpath Trend Analysis

The STA algorithm has the following three stages: (1) Preliminary Stage, (2) First Pass, and (3) Second Pass. These stages are briefly described below. The full description of the STA algorithm can be found in ( 8 ).

**Preliminary Stage.** The algorithm initially takes a series of fixations for each user on a web page and the AOIs of the page. It then finds the corresponding AOI for each fixation to represent the user scanpaths in terms of the AOIs. For example, the scanpath in Figure 1 is represented as MPPQ as the user fixated M, P, again P and Q respectively. The durations of these fixations are also stored.

**First Pass.** The aim of the first pass is to analyse the user scanpaths for the discovery of the trending AOIs based on the total number of fixations and the total duration of fixations (dwell time) on the AOIs. A user can fixate the same AOI more than once consecutively (such as, M**PP**Q) and/or non-consecutively (such as, M**P**Q**P**). We refer to each non-consecutive visit as an instance. For example, there are two instances of P in M**P**Q**P**. As the STA algorithm performs sequential analysis, these instances should be differentiated. To differentiate the instances of the same AOI, different numbers are assigned to them where the longest instance gets the first number. For example, if a user fixates M, M, P, M and M for 100 ms, 200 ms, 200 ms, 400 ms and 200 ms respectively, his/her scanpath is represented as M2 [100 ms] M2 [200 ms] P1 [200 ms] M1 [400 ms] M1 [200 ms] because the dwell time of the first instance of M (100 ms + 200 ms) is less than the dwell time of its second instance (400 ms + 200 ms).

When the algorithm differentiates the instances, it starts the discovery of the trending instances. When the total number of fixations on a particular instance is greater or equal to the minimum total number of fixations on the fully shared instances of the user scanpaths, and the dwell time on the instance is greater or equal to the minimum dwell time on the fully shared instances, the instance is defined as a trending instance. After the discovery of the trending instances, the algorithm removes other instances from the user scanpaths as they will not be in the trending path.

**Second Pass.** In the second pass, the user scanpaths are firstly collapsed by merging the same instances to determine the exact positions of the instances in the scanpaths because the second pass computes the sequential priority value for each instance in each scanpath by using the positions. For example, when the following scanpath is available M1 [150 ms] P1 [125 ms] P1 [150 ms] Q1 [125 ms], the positions of M1, P1 and Q1 should be zero, one and two respectively (the starting position is zero). When the algorithm merges the same instances, it combines them into one instance where its duration is equal to the total duration of the instances (such as, M1 [150 ms] P1 [125 ms] P1 [150 ms] Q1 [125 ms] → M1 [150 ms] P1 [275 ms] Q1 [125 ms]). The total number of fixations on each instance (i.e., the number of occurrence for each instance) is also stored. After that, the sequential priority value (ψ) of each instance in each user scanpath is computed with Equation (1) where P represents the instance position in the scanpath and L represents the length of the user scanpath. 1 and 0.1 are given the max and min values respectively to give 1 point the first instance and 0.1 points to the last instance.

**(1):**
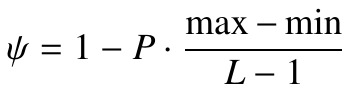


The total priority value (Ψ) for each instance is then computed with Equation (2) where n represents the number of user scanpaths. In particular, if there are five different instances in total (such as, M1, M2, P1, P2 and Q1), then the total priority value is calculated for each of them. The total priority values of the instances are then used by the algorithm to locate the instances in the trending path in descending order, thus the overall positions of the instances in the user scanpaths are preserved. If more than one instance share the same total priority value, their dwell time and the total number of fixations on the instances are also taken into consideration. In the end, the algorithm removes the numbers of the instances (such as, M1 → M), and then deletes the consecutive repetitions (such as, MPPQ → MPQ) to represent the trending scanpath in terms of the AOIs.

**(2):**
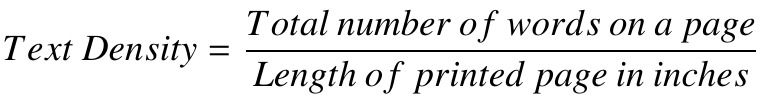


**Figure 3.**
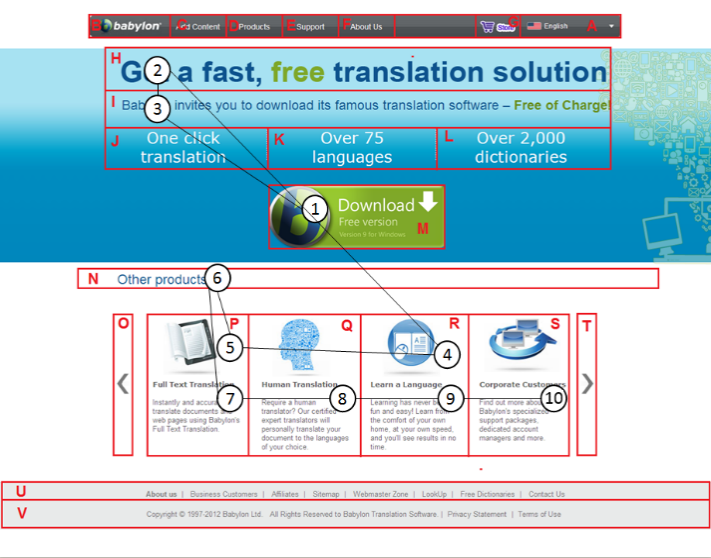
The trending scanpath on the Babylon web page

**Figure 4.**
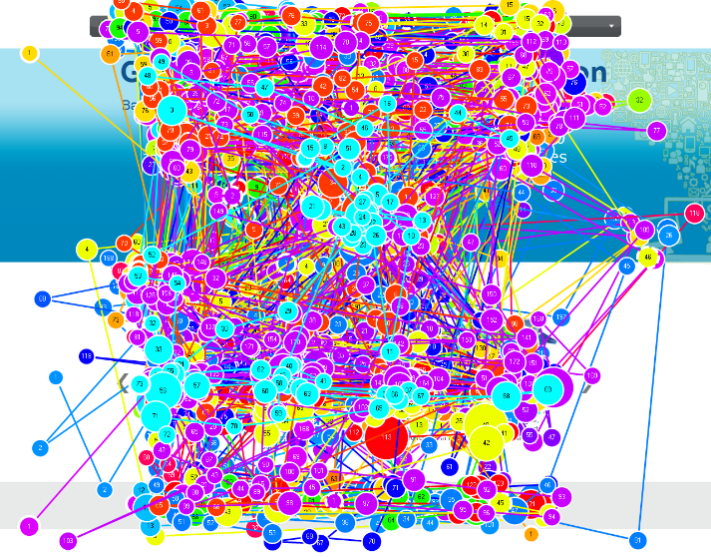
Multiple scanpaths on the Babylon web page

As an example, Figure 3 illustrates the trending scanpath on the Babylon page which was constructed with the STA algorithm by using the scanpaths in Figure 4. The trending scanpath shows us which AOIs are most commonly used and in which order. If we remove the unused AOIs and re-organise the remaining ones based on the trending scanpath, we would provide faster download of the page and direct access to the commonly used AOIs based on the desired order.

### Eye Tracking Study

To investigate whether the possibility of achieving almost the same results with fewer users in scanpath trend analysis ( 6 ) is restricted to a particular segmentation approach, we used the same dataset which is from an eye tracking study. This study is briefly described below and its full description can be found in ( 6, 8 ).

**Equipment.** Tobii T60 eye tracker was used to record the eye movements of the users. It was built-in a 17'' monitor and the screen resolution of the monitor was adjusted to 1280 x 1024.

**Pages.** The web pages used in the eye tracking study were randomly chosen from a group of pages used by Akpınar and Yeşilada ( 9 ). In their study, they investigated the visual complexities of the home pages of the top 100 websites listed by Alexa.com by using the ViCRAM tool ( 27 ), and then created three groups of pages based on their complexity (low, medium and high) where each group contains 10 randomly chosen pages. For this eye tracking study, two pages were randomly selected from each group: Apple & Babylon from the low complexity group, AVG & Yahoo from the medium complexity group, Godaddy & BBC from the high complexity group (see Appendix).

Some of the web pages contain much more text compared to others. For example, the Yahoo page contains much more text (353 words) compared to the Apple page (86 words). To quantitatively illustrate the text density of the pages, we referred to the text density function in Equation (3) given by Spool, Scanlon ( 28 ). Since the pages were shown in a fixed screen, we only report the total number of words seen by the participants on each page in Table 1.

**(3):**
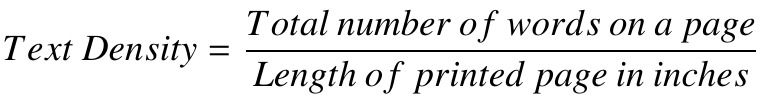


**Table 1.** The total number of words on a page

**Page**	**The total number of words on a page**
Apple	86
Babylon	156
AVG	162
Yahoo	353
Godaddy	163
BBC	300

**User Tasks.** The participants were requested to perform two different kinds of tasks on the web pages called browsing and searching tasks. For the browsing tasks, there was no specific objective, so the participants freely browsed on the pages. For the searching tasks, the participants needed to locate some specific information or items on the web pages. The searching tasks used in the eye tracking study are provided in Table 2.

**Table 2.** The searching tasks used in the eye tracking study

**Page**	**Tasks**
Apple	(a) Can you locate a link which allows watching the TV ads relating to iPad mini? (b) Can you locate a link labelled iPad on the main menu?
Babylon	(a) Can you locate a link that you can download the free version of Babylon? (b) Can you find and read the names of other products of Babylon?
AVG	(a) Can you locate a link which you can download a free trial of AVG Internet Security 2013? (b) Can you locate a link which allows you to download AVG Anti-virus Free 2013?
Yahoo	(a) Can you read the titles of the main headlines which have smaller images? (b) Can you read the first item under the News title?
Godaddy	(a) Can you find a telephone number for technical support and read it? (b) Can you locate a text box where you can search for a new domain?
BBC	(a) Can you read the first item of the Sport News? (b) Can you locate the table that shows market data under the Business title?

**Procedure.** The study was performed in a quiet room. Before the participants viewed the web pages, they read an information sheet about the study and singed a consent form. Their gender, age-group and education level were then asked. The participants were also asked to rank the web pages based on their usage (Daily, Weekly, Monthly, Less than once a month, Never). After that, they viewed the web pages twice in a random order with counterbalancing for the browsing (30 seconds) and searching tasks (max 120 seconds). For example, one participant completed the tasks in the following order: “Babylon - Search, AVG - Search, Apple - Browse, Godaddy - Search Yahoo - Browse, Babylon - Browse, Apple - Search, Yahoo Search, Godaddy - Browse, AVG - Browse, BBC Browse and BBC – Search” and the other one completed in this order: “BBC - Search, Babylon - Browse, BBC Browse, Babylon - Search, AVG - Search, Godaddy Browse, AVG - Browse, Apple - Search, Yahoo Browse, Yahoo - Search, Apple - Browse, Godaddy Search'”. The participants could be familiar with the pages during their first visits and this situation could affect how they interact with the pages during their second visits. Hence, for each web page, one half of the participants firstly completed the searching task and then the browsing task whereas another half firstly completed the browsing task and then the searching task. The tasks were read by the researcher to the participants when the relevant pages were shown on the screen during the eye tracking sessions. The participants were not allowed to use a keyboard and a mouse as the tasks could be completed by only scanning the pages. When they completed their eye tracking sessions, they were asked to draw what they remember on the pages. Apart from the fixations made by the participants after completing the searching tasks, none of the fixations were excluded from the analysis.

**Participants.** The eye tracking study conducted with 81 users at two different universities with 40 female and 41 male users. Most of these users were students from the universities and they were mainly between the ages of 1834. The pages used in this study were not regularly visited by the users. The percentage of the users who reported that they never visited the pages or visited them less than once a month were as follows: Apple: 71.6%, Babylon: 93.8%, AVG: 92.6%, Yahoo: 61.7%, Godaddy: 98.8% and BBC: 46.9%.

### Page Segmentation Approaches

Web pages can be segmented automatically or manually based on the goals of studies ( 7 ). In the previous study to investigate the possibility of achieving almost the same results with less users in scanpath trend analysis, the experiments were conducted with the extended and improved version of the VIPS approach to automatically segment the pages ( 6, 9 ). In this current paper, we used the BOM approach for the automatic segmentation of the web pages ( 12 ). Similar to the VIPS approach, the BOM approach also uses the DOM structure and the visual representation of web pages but it computationally applies different algorithms. Specifically, in the BOM approach, the segments are also grouped based on the four Gestalt laws (Proximity, Similarity, Closure and Simplicity). Both of the segmentation approaches have also a granularity level parameter which affects the size of segments and the number of segments. In our experiments, we used the BOM approach with its default value (0.3) for this parameter.

**Figure 5.**
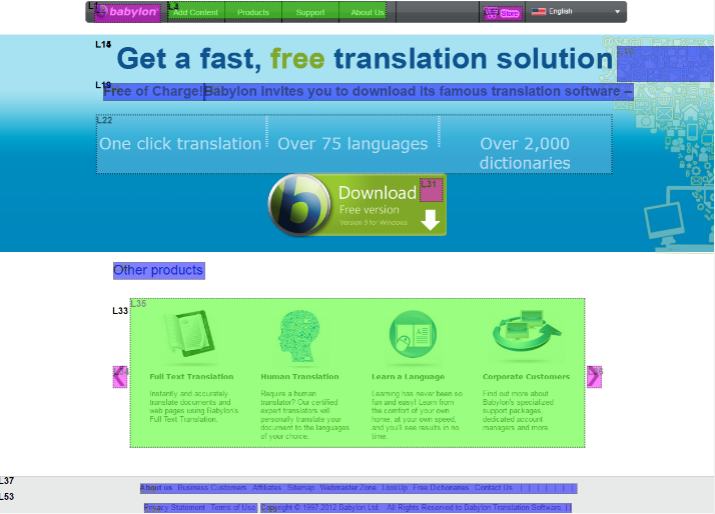
The Babylon page segmented by the current publicly available implementation of the BOM approach

**Figure 6.**
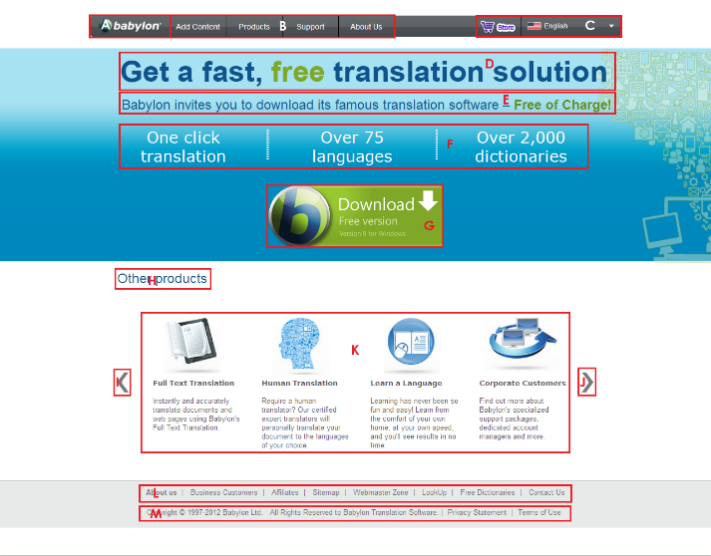
The Babylon page segmented by the BOM segmentation approach [The segmentation problems highlighted in Figure 5 are addressed in this version]

The current publicly available implementation of the BOM approach ( http://www-poleia.lip6.fr/~sanojaa/BOM/ )could not properly segment some of the web pages. Thus, we followed a systematic approach to fix the problems ( 29 ) by considering the algorithm given in their papers ( 12, 30 ): (1) If an element covered only space, the element was ignored. (2) If a larger element covered other smaller elements and not all parts of this larger element were covered by the smaller elements, the larger element was used. This was to ensure that all parts of the larger element are covered by an element. (3) If there were overlaps between multiple elements, the borders of the elements were adjusted to deal with the overlaps. (4) If there was an element which was not located in any element as a result of the segmentation, it was located in the nearest element. As an example, Figure 5 shows how the Babylon page segmented by the current publicly available implementation of the BOM approach. The menu item for the language selection was not covered by any block, and therefore it was located in its nearest block L13 (The menu item “Store”). Figure 6 shows how the Babylon page was divided into their elements by using the BOM approach with a systematic approach for fixing the problems.

We also followed a systematic way to find other automatic page segmentation approaches with a public implementation by looking at the relevant papers and searching from the web. As illustrated in Table 3, other segmentation approaches do not have their public implementations and/or they are not applicable (NA) for this study. If we could not find the implementation of the proposed approach, we sent an email to the author(s) to ask whether they could provide us the implementation of their proposed segmentation approach. If the authors did not respond to our email, we assume that their segmentation approaches do not have a public implementation. Moreover, not all segmentation approaches are suitable for our study. In particular, if a segmentation approach focusses on getting specific parts of web pages, it is considered as not applicable (NA) for our study as we are not interested in only specific parts of web pages. For example, one approach focusses on getting the main content of a web page instead of segmenting the web page into its visual elements ( 31 ). There are also some approaches which are outdated such as the approach of MilicFrayling and Sommerer ( 32 ) which focusses on HTML table tags. These approaches are also classified as NA. Although the page segmentation approach of Michailidou, Harper ( 33 ) is publicly available, it has some bugs and does not generate a proper segmentation.

**Table 3.** The public availability of the page segmentation approaches [+: Available, -: Not Available, NA: Not Applicable] - The references are in ( 7)

**Category**	**Source**	**Availability**
Clustering	Alcic and Conrad (2011)	-
Custom	Cheng and Gotz (2009)	NA
	Yang and Shi (2009) & Xiang et al. (2007)	-
	Guo et al. (2007)	-
	Gupta et al. (2007)	NA
	Hattori et al. (2007)	-
	Gu et al. (2002)	-
	Chen et al. (2001)	NA
	Sanoja and Gançarski (12) & Sanoja and Gançarski (30)- BOM	+
	Wei, Lu (34)	-
	Zeleney, Burget (35)	-
DOM-Based	Fauzi et al. (2009)	NA
	Vineel (2009)	-
	Xiao et al. (2008)	-
	Chen et al. (2005, 2003)	-
	Yin and Lee (2005)	-
	Liu et al. (2004)	-
	Lin and Ho (2002)	NA
Heuristics	Toh and Hong (36)	-
	Burget and Rudolfova (2009)	NA
	Burget (2007)	
	Ahmadi and Kong (2008)	-
	Michailidou, Harper (33)	+
	Kreuzer, Hage (37)	NA
	Debnath et al. (2005)	-
	Kovacevic et al. (2002)	-
	Milic-Frayling and Sommerer (32)	NA
Image Processing Algorithms	Cao et al. (2010)	-
Machine Learning	Chakrabarti et al. (2008)	-
	Borodin et al. (2007)	NA
	Mahmud et al. (2007)	NA
	Baluja (2006)	-
	Bing, Guo (38)	NA
	Feng, Zhang (39)	-
Pattern Matching	Xiang and Shi (2006)	-
	Nanno et al. (2004)	-
	Cuzzolaa, Jovanović (40)	-
	Whang et al. (2001) & Hwang et al. (2003)	NA
Ranking-Based	Yin and Lee (2004)	NA
Text-Based	Sun et al. (2011)	NA
	Kohlschütter, Fankhauser (31)	NA
	Kohlschütter (2009)	NA
	Kohlschütter and Nejdl (2008)	-

**Figure 7.**
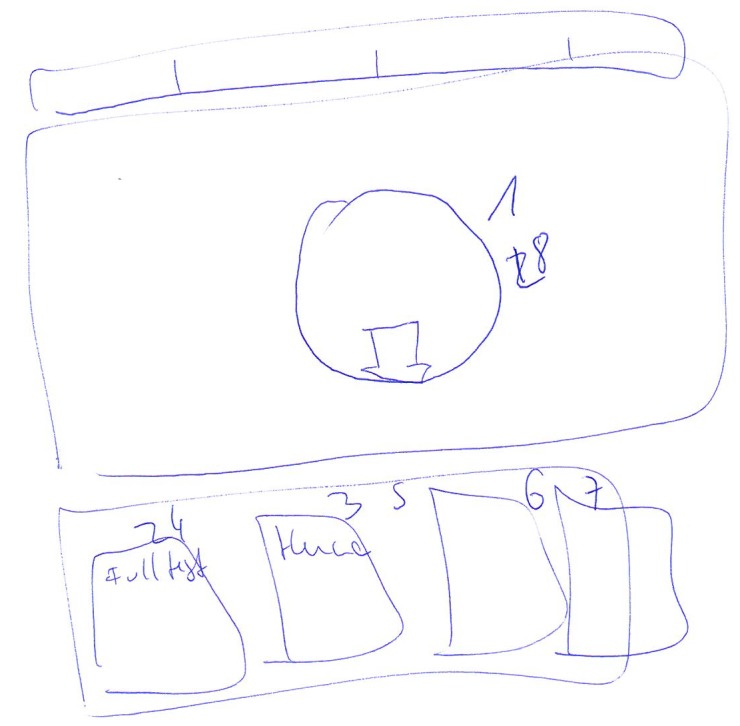
The Babylon page drawn by the participant 1

**Figure 8.**
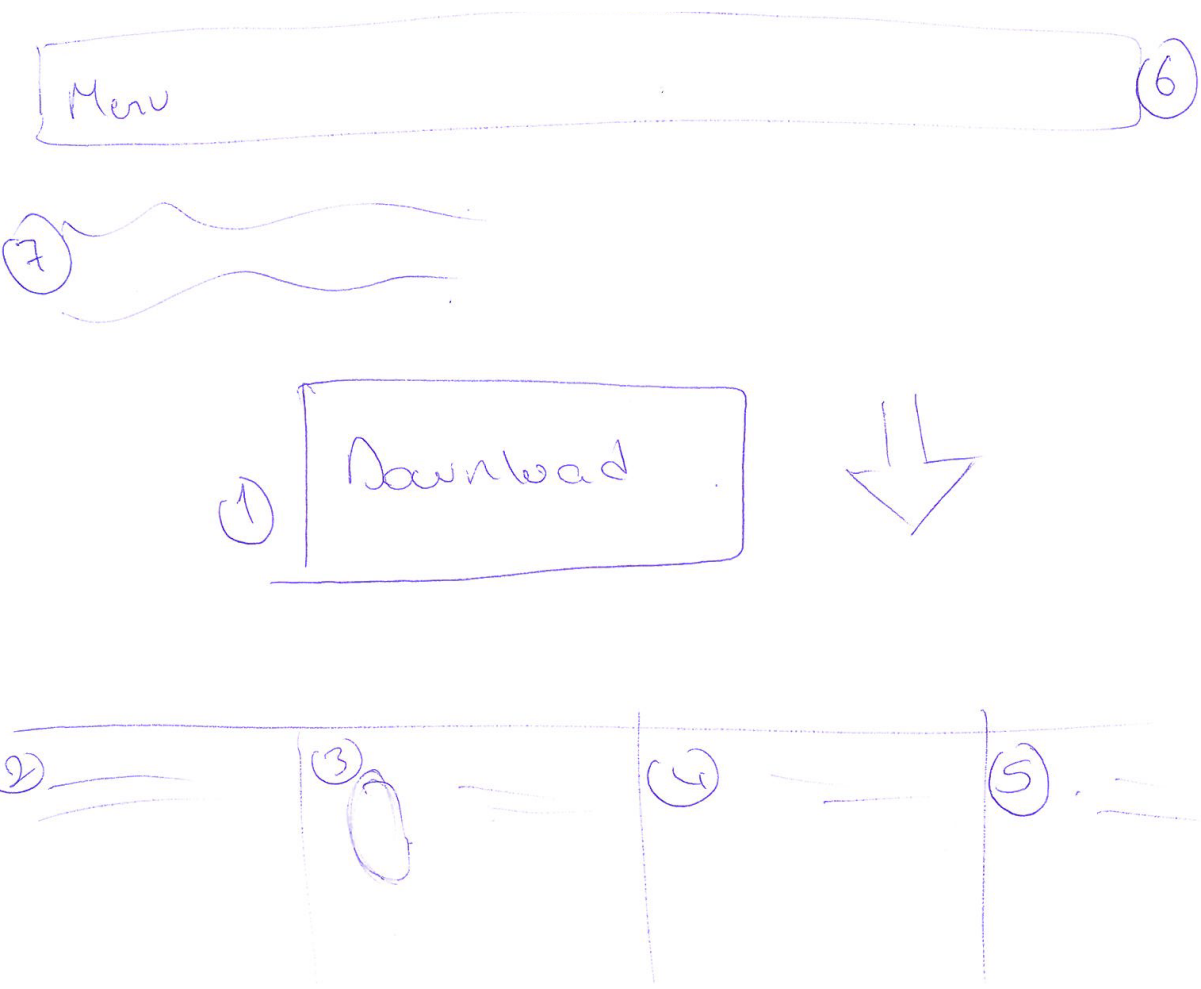
The Babylon page drawn by the participant 2

**Figure 9.**
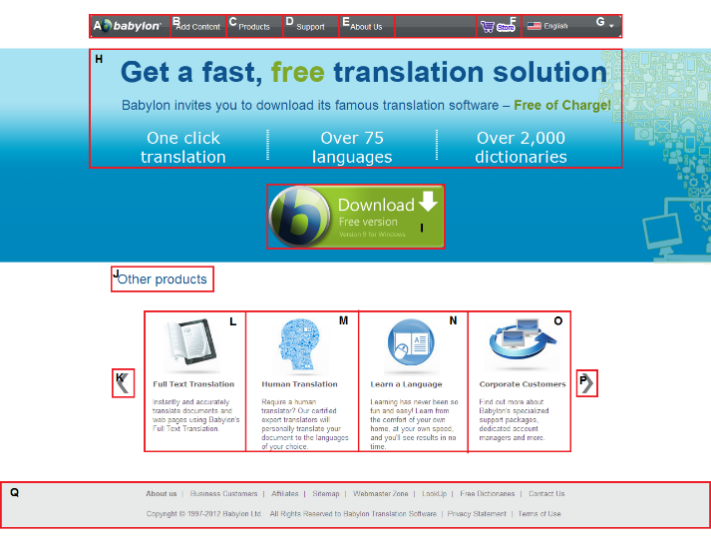
The Babylon page segmented by the user driven segmentation approach

We also manually segmented the web pages as manual segmentation can also be preferred by researchers based on their goals ( 13 ). In order to be more objective, we did not want to do the segmentation by ourselves, and therefore we used the recall approach. At the end of the eye tracking study described above, the participants were asked to draw what they remember about the layouts of the web pages on a piece of paper after they completed their tasks on the web pages. Figure 7 and Figure 8 shows how the Babylon web page was drawn by two participants. We systematically combined their drawings to identify the AOIs of the web pages. To be more objective, three researchers firstly combined the drawings for each web page, including other researchers who are not authors of this paper. For example, they noted how many users divided the main menu into its elements and how many of users drew the main menu as an entire element. Once they combined the drawings, their notes were analysed to see all the elements drawn by the users. If there was a larger element that covers other smaller elements, the smaller elements were used as they were more specific. For example, if a menu was divided into their items, the menu items were considered as separate elements instead of using the menu as one element. Even though we applied the same approach here as explained for the BOM approach ( 12 ) (If a larger element covered other smaller elements and not all parts of this larger element were covered by the smaller elements, the larger element was used), we used the smaller elements since all parts of the larger element were covered by the smaller elements here. As an example, Figure 9 shows how the Babylon page is segmented by using this approach.

The overview of the differences between the segmentations of the VIPS, BOM and user driven approaches in terms of the number of AOIs generated and their coverage on the web pages can be seen from Table 4. Some parts of a particular web page may be located in separate AOIs with some segmentation approaches whereas the same parts may be located in only one AOI with other approaches. For example, each of the other Babylon products (“Full Text Translation”, “Human Translation”, “Learn a Language” and “Corporate Customers”) was located in a separate AOI with the VIPS (with its most satisfactory granularity ( 9 )) and user driven segmentation approaches (see Figure 1 and Figure 9) however all these products are located in the same AOI with the BOM segmentation approach (with its default granularity parameter) (see Figure 6). As illustrated in Table 4, the VIPS approach provides more and smaller segments on the Babylon, AVG, and BBC web pages in comparison with the BOM and user driven approaches. In contrast, it provides fewer and larger segments on the Yahoo page.

**Table 4.** The % of the coverage of the AOIs generated by the VIPS, BOM and user driven approaches [N: The number of AOIs, M: Mean, MD: Median, SD: Standard Deviation]

**Page**	**Approach**	**N**	**M**	**MD**	**SD**	**Max**	**Min**
Apple	VIPS	18	2.68	0.82	5.46	23.57	0.06
	BOM	7	7.54	3.38	9.57	26.58	0.77
	User driven	18	2.58	0.76	4.84	21.03	0.30
Babylon	VIPS	22	1.98	1.60	1.65	5.27	0.24
	BOM	13	2.79	1.05	4.39	16.09	0.06
	User driven	17	2.62	0.56	3.98	15.58	0.14
AVG	VIPS	25	1.97	0.45	4.65	20.93	0.07
	BOM	7	6.99	1.82	8.16	21.05	0.32
	User driven	20	2.41	2.47	1.90	8.35	0.44
Yahoo	VIPS	10	6.93	1.94	9.57	25.78	0.19
	BOM	12	5.09	1.56	7.13	24.78	0.08
	User driven	24	2.57	0.92	2.88	9.14	0.10
Godaddy	VIPS	16	4.16	0.52	9.44	35.15	0.10
	BOM	11	5.64	1.46	7.21	18.42	0.11
	User driven	19	2.94	1.18	4.36	17.61	0.35
BBC	VIPS	21	3.22	0.95	4.26	12.90	0.15
	BOM	5	14.43	8.79	16.28	43.22	4.24
	User driven	10	6.72	6.62	3.85	14.45	0.17

The segmentation of the pages with each of these segmentation approaches is visualised and provided in our external repository. Once the web pages were segmented by using both the BOM segmentation approach ( 12 ) and the user driven approach, we replicated the experiments of our previous study ( 6 ) by using the same methodology as described in the following section.

### Analysis Procedure

As some of the users could not successfully complete their tasks on some of the web pages and/or experienced some eye calibration problems, they were classified as unsuccessful users and excluded from the experiments. If a particular user was unsuccessful on the page X but successful on the page Y, s/he was excluded from only the page X. Due to this reason, the number of users on the web pages for the browsing and searching tasks were not the same. However, it would be better to have the same number of users on all the pages for both types of the tasks for the consistency among the pages and estimation of the overall graph. Thus, some of the successful users were also randomly excluded from the experiments. As a result, the experiments were conducted with 65 users on each web page for the browsing and searching tasks.

The STA algorithm was then applied to the users to discover their trending scanpaths on the pages which were segmented by using the BOM approach ( 12 ) and the user driven approach. Following this, smaller groups of users were chosen with the size of one user to 64 users from the successful users and then the STA algorithm was applied to these groups to discover their trending scanpaths. In other words, sub-groups were randomly created with different sizes from 1 user to 64 users and their trending scanpath were identified with the STA algorithm (i.e., group of one user, group of two users, group of three users … group of 64 users). The results of the smaller groups were then compared with the results of the entire group. To deal with any possible effects of the selected users, 100 different combinations were generated for each group size and their median similarity was taken as a similarity to the entire the group to counteract extreme cases ( 18 ).

To compare two scanpaths, the Levenshtein Distance (String-edit) algorithm was used as it has widely been used to determine the distance between two scanpaths ( 41, 42 ). This algorithm transforms one scanpath to another by using the minimum number of addition, deletion and substitution operations. The minimum number of operations shows the distance between the two scanpaths. For instance, the distance between DEFG and DEHG is equal to one because it is sufficient to substitute F with H to transform one of them to another or vice versa.

The sizes of AOIs and the distances between the AOIs are potentially different, as a consequence the substitution costs between all pairs of AOIs may not be the same ( 41 ). A substitution matrix has been suggested to take this issue into account ( 42 ). This matrix should include substitution costs between all pairs of AOIs. As suggested by Takeuchi and Habuchi ( 42 ), the Euclidean distances between the AOIs were used to generate a substitution matrix in the experiments. Equation (4) below illustrates how to calculate a substitution cost between two AOIs *I* and *J* based on the Euclidean distance where *I₁* and *I₂* are x and y coordinates of the centre of the element *I* and α is a type of normalisation parameter ( 42 ). The normalisation parameter was taken as 0.001 ( 42 ). The matrix was then integrated into the String-edit algorithm to determine the distance between two scanpaths by computing the minimum cost for transforming one scanpath to another.

**(4):**
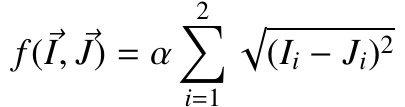


To find a similarity between two scanpaths as a percentage based on the String-edit distance ( 43 ), the distance (*D*) between two scanpaths was firstly divided by the length of the longer scanpath (*L*) to calculate a normalised score to prevent possible inconsistencies that can be caused by different lengths. The normalised score was secondly subtracted from one and finally multiplied by 100. The relevant formula is shown in Equation (5). For example, the similarity between DEFG and DEHG is calculated as 75% as the distance (*D*) between these scanpaths is equal to one (the substitution between F and H), and the length of the longer scanpath (*L*) is equal to 4 (both of them have the same length in this example).

**(5):**
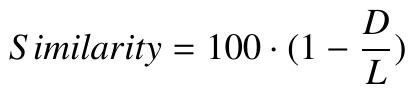


As we investigated how many users are needed to achieve almost the same results as 65 users, our dependent variable was the number of users and our independent variable was the similarity to the results of 65 users. Once we calculated the similarity to the entire group for each smaller group, the curve estimation feature of SPSS ( http://www.ibm.com/analytics/us/en/technology/spss/ ) was applied to select the best curve that fits into the mean of these similarities on the six web pages for the browsing and searching tasks. Specifically, the curve with the minimum standard error of the estimate was selected.

## Results

**Figure 10.**
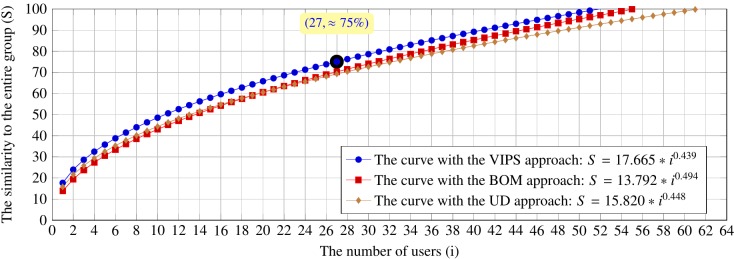
The estimated curves by using the VIPS, BOM and user driven segmentation approaches on the six web pages for the searching tasks

**Figure 11.**
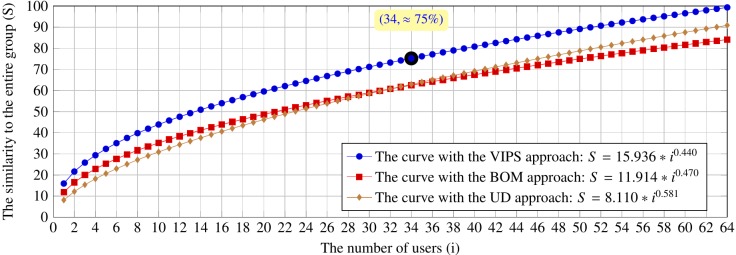
The estimated curves by using the VIPS, BOM and user driven segmentation approaches on the six web pages for the browsing tasks

Figure 10 and Figure 11 show the estimated curves by using the VIPS ( 9 ), BOM ( 12 ) and user driven segmentation approaches on the six web pages for the browsing and searching tasks with their equations where S is the similarity to the entire group and i is the number of users ( 6 ). The standard errors of the estimated curves for the VIPS, BOM and user driven approaches are 0.073, 0.066 and 0.034 for the searching tasks, and 0.044, 0.063 and 0.082 for the browsing tasks respectively.

To compare two estimated curves, the absolute difference in the curves was calculated for each group size as illustrated in Table 5. For instance, the difference between the curves generated by using the VIPS approach and the BOM approach for three users is equal to 5.87 (With the BOM: 19.97, With the VIPS: 25.84) for the browsing tasks and 4.88 (With the BOM: 23.73, With the VIPS: 28.61) for the searching tasks.

**Table 5.** The differences between the curves generated by using the VIPS approach and the BOM approach where i is the number of users.

**Task**	**i**	**With the BOM**	**With the VIPS**	**Difference**
Browsing	1	11.91	15.94	4.02
	2	16.50	21.62	5.12
	3	19.97	25.84	5.87
	4	22.86	29.33	6.47
	5	25.38	32.35	6.97
	…	…	…	…
Searching	1	13.79	17.67	3.87
	2	19.42	23.95	4.52
	3	23.73	28.61	4.88
	4	27.36	32.47	5.11
	5	30.54	35.81	5.26
	…	…	…	…

Table 6 also illustrates the mean, median, maximum, minimum and standard deviation (SD) of the differences between the curves generated by using the VIPS, BOM and user driven approaches. As illustrated in the table, the mean difference between the curves generated by using the VIPS approach and the BOM approach is only 4.13% (SD: 1.13%) for the searching tasks and 11.90% (SD: 2.76%) for the browsing tasks. Furthermore, the mean difference between the curves generated by using the VIPS approach and the user driven approach is only 5.88% (SD: 1.47%) for the searching tasks and 11.49% (SD: 1.62%) for the browsing tasks. Finally, the mean difference between the curves generated by using the BOM approach and the user driven approach is only 2.50% (SD: 1.68%) for the searching tasks and 3.23% (SD: 1.83%) for the browsing tasks.

**Table 6.** The mean (M), median (MD), maximum, minimum and standard deviations (SD) of the differences between the curves generated by using the VIPS approach, the BOM approach and the user driven approach where i is the number of users

**Task**	**Approaches**	**M**	**MD**	**Max**	**Min**	**SD**	
Browse	VIPS-BOM	11.90	12.60	15.27	4.02	2.76	
	VIPS-User driven	11.49	11.85	13.37	7.83	1.62	
	BOM-User driven	3.23	3.39	6.94	0.00	1.83	
Search	VIPS-BOM	4.13	4.33	5.53	1.96	1.13	
	VIPS-User driven	5.88	6.22	7.75	1.85	1.47	
	BOM-User driven	2.50	2.08	5.79	0.04	1.68

To sum up, the previous and current results are mainly consistent with ~5\% difference for the searching tasks and ~10\% difference for the browsing tasks.

## Discussion

Based on the results presented in this paper, we can suggest that we can see almost the same trend in the curves when we use the VIPS ( 9 ), BOM ( 12 ) and user driven approaches to segment the web pages. In particular, the results obtained from the BOM and the user driven approaches are very close to each other. Therefore, we can suggest that the previous and current results are consistent with some small deviations (~5\% difference for the searching tasks and ~10\% difference for the browsing tasks).

Although the VIPS approach is popular among researchers to segment web pages, they can also use other page segmentation approaches. However, the previous study did not show whether the possibility of achieving almost the same results with fewer users is limited to the VIPS approach. Therefore, when researchers want to use another segmentation approach with the STA algorithm, they could not ensure whether the possibility is still valid for them. Hence, this study is beneficial for them from this aspect as it shows that we can approximate almost the same results with a smaller group of users in scanpath analysis regardless of the segmentation approach used.

As mentioned in our related work section, although other aspects of eye tracking data (especially, heat maps ( 25 )) have been studied, the number of users required for scanpath analysis has not been studied in depth. The possibility of achieving almost the same results with less users in scanpath trend analysis has been raised but the segmentation effect on this possibility was not investigated. Therefore, this study is an important step forward in existing research. Furthermore, the results are still consistent with the suggestions for heat maps ( 25), especially for the browsing tasks, even though the analysis of heat maps are much easier in comparison with scanpath analysis (see Figure 2 and Figure 4).

We also observed some differences in the curves generated by using the VIPS, BOM and user driven segmentation approaches. Specifically, the VIPS approach has higher similarities compared to other two segmentation approaches, especially for the browsing tasks. The differences in the curves generated for the browsing tasks are higher in comparison with the differences in the curves generated for the searching tasks. Since the participants did not require to locate specific items on the web pages for the browsing tasks and they were allowed to browse freely, the variations between the user scanpaths tend to be higher, and therefore it is quite normal to see higher difference in the curves generated for the browsing tasks. We think that the differences in both the searching and browsing tasks might be caused by the sizes of the AOIs. In particular, when the BOM approach is used to automatically segment the web pages, fewer and larger AOIs are usually generated. Therefore, user scanpaths become shorter, as a consequence their resulting paths also become shorter. In these cases, small deviations between two scanpaths considerably decrease the similarity between the scanpaths. For example, the similarity between the scanpaths ABC and ACB is calculated as 33.33% by the standard String-edit algorithm, even though they have the same elements ( 41 ). To investigate this expectation, we should conduct further experiments as we cannot say that the VIPS approach creates smaller blocks in all the cases in comparison with other approaches. In the further experiments, the same methodology can be applied by segmenting web pages with different granularity levels and the effects of the AOI sizes can be investigated. The reason for the VIPS approach having higher similarities could also be that it might provide the most similar AOIs to the AOIs which were actually used by the participants. However, this possible reason should be further investigated by determining the similarities between the AOIs generated by the segmentation approaches and the AOIs which were actually used by the users. However, in order to do this, a systematic approach should firstly be developed to discover the borders of the AOIs which were actually used. These borders can then be used for analysis

A study conducted by Akpınar and Yeşilada ( 9 ) suggests that more and smaller segments are preferred by users instead of fewer and larger segments for the VIPS approach. Therefore, in the previous study ( 6 ), the experiments was conducted by using the suggested granularity level of the VIPS algorithm. However, in our current experiments, we had to use the default granularity level of the BOM approach as no specific suggestion is provided. Since the granularity level affects the segmentation, it may also affect the results. Therefore, when a segmentation approach is proposed, its most successful/preferable/satisfactory granularity level should also be provided, if appropriate.

**Figure 12.**
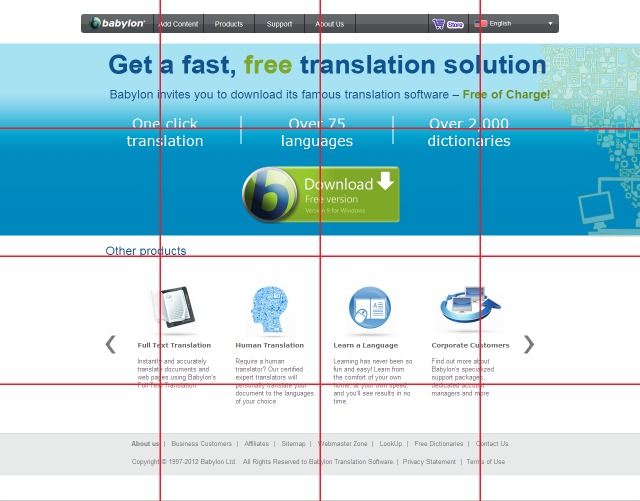
The Babylon page with a 4-4 grid segmentation.

Because of the limited number of publicly available automatic segmentation approaches ( 7 ), we could only use the BOM approach for automatically segment the web pages to cross-check the previous findings obtained by using the VIPS approach. Since web pages can also be manually segmented by researchers based on their goals ( 13 ), we also applied a user driven approach to manually segment the web pages. However, further experiments need to be conducted by using more segmentation approaches and different eye tracking datasets with the same methodology to increase the generalizability of the probability of accessing almost the same results with fewer users. For example, it would be worthwhile to investigate how this possibility is affected when all users are familiar with web pages or when web pages are segmented into very small or very large elements. Furthermore, a number of eye tracking experts could also be invited to discuss and identify the AOIs of the web pages based on their experience and then the same methodology could be applied. However, manual segmentation is typically based the goals of the study and it is almost impossible to estimate all possible segmentations. We could also apply the grid segmentation. However, the elements of the web pages could be inappropriately divided in that case as the web pages were not necessarily designed with a grid layout. For example, Figure 12 shows how the Babylon page could be segmented with a 4x4 grid segmentation. As illustrated, some elements are inappropriately divided, such as the text under the “Full Text Translation” title. Therefore, we did not use the grid segmentation in our study.

In this study, only the STA algorithm was used but the methodology can also be applied to other scanpath analysis algorithms to investigate the effects of the number of users on their results. For example, the SPAM (Sequential Pattern Mining) algorithm was used by Hejmady and Narayanan ( 44 ) to discover visual attention patterns during program debugging with an IDE (Integrated development environment). They conducted their study with 19 participants but they did not know how their results could be affected if they had less or more participants. As it is more reliable to report stable results, it is worthwhile to investigate the effects on the number of users on other existing scanpath analysis algorithms.

This study in not without limitations. The home page of six web pages were used. Although these web pages had different levels of visual complexities, more web pages with more complicated and different designs would be better to increase the generalizability of our findings. In this paper, we provided the results of the six web pages together as illustrated in Figures 10 and 11. However, the visual complexity and text density may affect the results. If we had more web pages in each visual complexity group (low, medium and high), we would be able to investigate whether the visual complexity of web pages affects the possibility of achieving almost the same results with a less number of users. We currently have two web pages in each visual complexity group. Thus, if we try to investigate the effects of the visual complexity, we will have only two pages in each visual complexity group and therefore the results would not be representative. Likewise, if we try to investigate the effects of the text density, we will have only one page with lower density (Apple: 86 words) and two pages with higher density (Yahoo: 353 word, BBC: 300 words), and therefore the results would not be conclusive. However, it would be interesting to investigate the effects of the visual complexity and text density on the possibility of achieving almost the same results with a less number of users. We are planning to explore these possible effects in the future studies. One can design a study to take into consideration both visual complexity and text density as features in selecting web pages

Most of the participants were university students and daily web users. Even though there were many participants with different backgrounds, the increase in the diversity of the users would be better to draw stronger conclusions. Furthermore, we only used the Levenshtein Distance algorithm with a substitution cost matrix to compute similarities between scanpaths. Albeit the Levenshtein Distance algorithm has widely been used in eye tracking research, other similarity measures, such as Needleman and Wunsch ( 45 ), can also be used in the future and the findings can be cross-checked.

To sum up, this work is a step forward for better understanding of how the number of users affect scanpath analysis. It would benefit to both eye tracking and human computer interaction (HCI) researchers to estimate the ideal sample size for their studies by considering their time and budget when they want to conduct scanpath trend analysis. The methodology used is also promising for researchers who developed a scanpath analysis algorithm and want to investigate the effects of the number of users on the results of their algorithms.

## Conclusion

In this paper, we show that we can approximate almost the same results with a smaller group of users in scanpath trend analysis regardless of the segmentation approach used. The current findings concur with the findings of Eraslan, Yesilada ( 6 ) suggesting that it is possible to achieve almost the same results with fewer users. Specifically, the findings of Eraslan, Yesilada ( 6 ) suggest that it is possible to achieve 75% similarity to the results of 65 users with 27 users for searching tasks and 34 users for browsing tasks. In this paper, we investigated both the automatic and manual segmentation effects on the findings. Based on our experiments, we can suggest that our current findings are mainly consistent with the previous findings.

Although it might not be possible to provide exactly the same results with fewer users, 75% similarity is a promising value for practitioners because it means that approximately 3/4 of the results are the same. For example, the similarity between the scanpaths ABCDBADBDEFE and ABCEBADCDEF is calculated as 75% with the standard String-edit algorithm ( 41 ). The practical usefulness of 75% and other similarities will be investigated in the future. This paper allows practitioners to know more about possible differences when they have lower sample size, thus it helps them to determine their sample size based on their time and the budget of their studies.

### Ethics and Conflict of Interest

The authors declare that the contents of the article are in agreement with the ethics described in http://biblio.unibe.ch/portale/elibrary/BOP/jemr/ethics.html and that there is no conflict of interest regarding the publication of this paper.

### Acknowledgements

The dataset used is from our eye tracking study that was approved by the Ethics Committee of School of Computer Science at the University of Manchester (approval ID: CS90). We would like to thank all our participants for their time and effort.

## Figures and Tables

**Figure 13.**
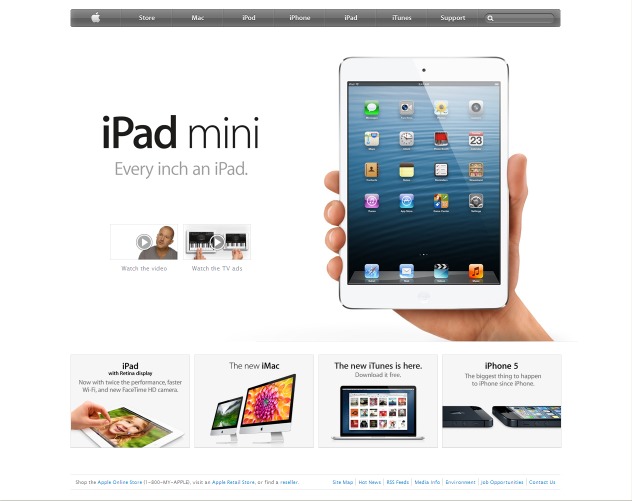
The Apple web page

**Figure 14.**
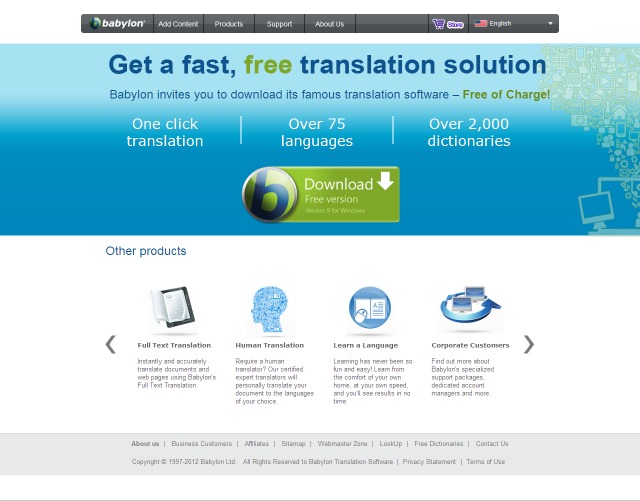
The Babylon web page

**Figure 15.**
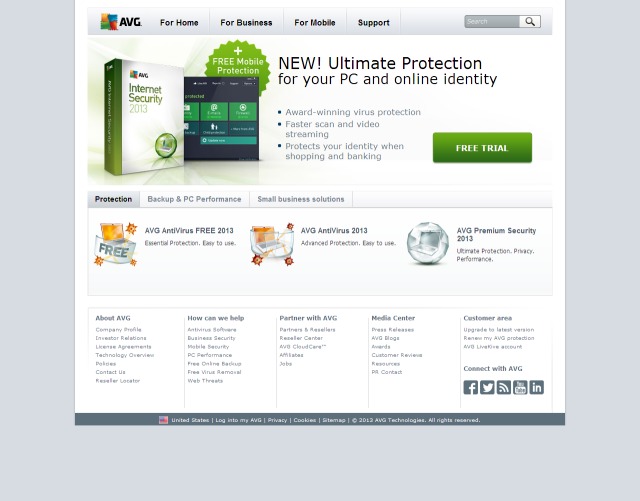
The AVG web page

**Figure 16.**
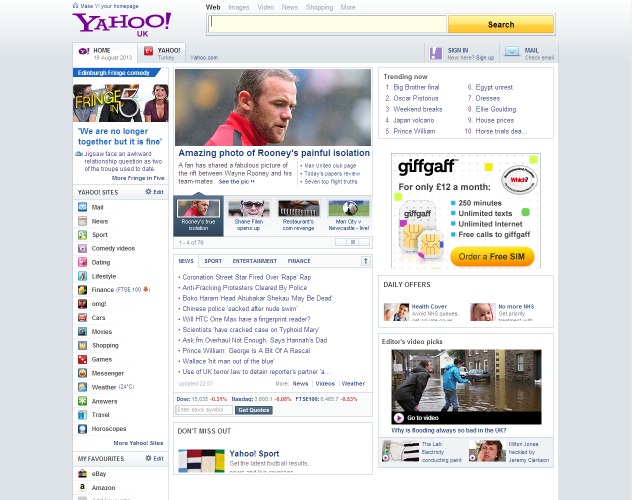
The Yahoo web page

**Figure 17.**
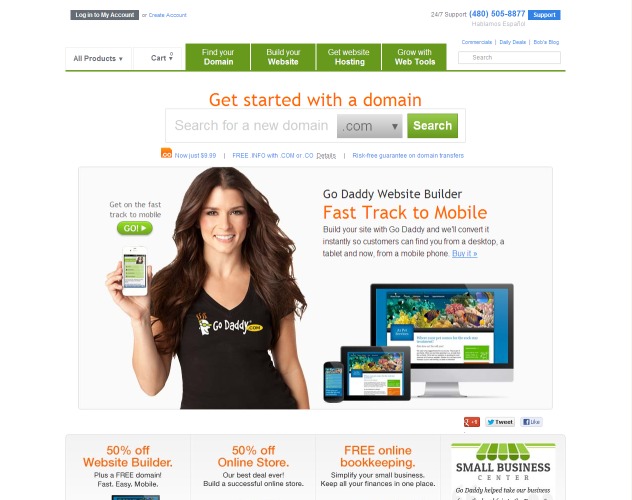
The Godaddy web page

**Figure 18.**
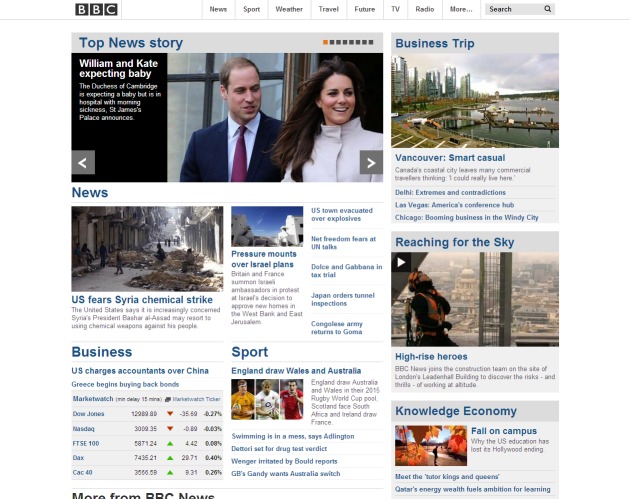
The BBC web page
